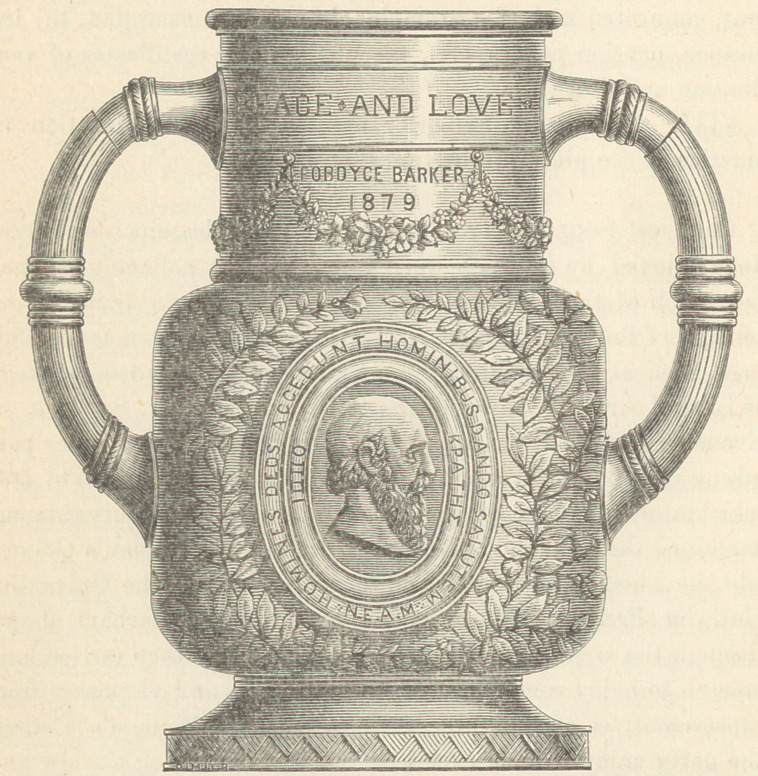# Mrs. Astor’s Present to the New York Academy of Medicine

**Published:** 1880-04

**Authors:** 


MRS. ASTOR'S PRESENT TO THE NEW YORK ACADEMY OF MEDICINE.—On the 15th of January last, Mrs. Augusta Astor presented to the New York Academy of Medicine, a costly and beautiful gift, in the form of a "loving cup." We are indebted to the kindness of the publishers of the N. Y. *Medical Record* for the annexed figure, which well represents the artistic finish of the original.

We have not reproduced this illustration without a purpose. When the wealthy and cultivated citizens of our great metropolitan .centers are ready to exhibit their sense of what the medical organizations in those centers are actually accomplishing, when they learn to appreciate the character of their representative men and are prepared to give evidence of that appreciation in their generosity, that is a good day for the citizens of any metropolis. Medical men in America are by no means the "social Pariahs" which our English friends have lately been called by one of their own number. They are in no sense, either beggars or paupers of the commonwealth. Industry, sobriety and even an ordinary degree of skill in the performance of their professional duties, are rewarded here with that comfortable and competent income which is far removed from the average of the immense majority of men.

The medical men of our land, and the medical institutions they have in charge, deserve well of the wealthy and cultivated classes. The incalculably large measure of charitable work performed by the profession in this country, probably represents a greater amount of unpaid labor than is to be found in any other field of human activities. The recognition of this, on the part of those who, whatever their wealth or social position, cannot fail to be indirectly benefited by it, is to us, a touching and grateful as well as graceful tribute. When some of the professorships in our medical colleges are properly endowed, and the scientific associations of medical men, are made the recipients of bequests intended to enlarge their powers of usefulness and their influence among all classes of society, then we shall have the solution of several problems which are now vexing the medical world.

We congratulate our brethren in New York, far more upon this evident recognition of their position as Academicians, than upon the gift they have received, costly and elegant though it be. And in reproducing the beauty of its exterior in these pages, we are not without the hope, that the time is near when some of the several medical associations of this city, shall in their own fireproof place of meeting, be made the recipient of a gift, which, even though it be not a "loving-cup," shall help to unite their members in the sympathy begotten of an earnest effort in an honorable and common cause.

---

IF any of our subscribers can, conveniently, send to the address of Dr. E. L. Holmes, 119 South Clark st., copies of the CHICAGO MEDICAL JOURNAL for November, 1866, and for August, 1867, they will confer a great favor.

## Figures and Tables

**Figure f1:**